# The Remission of Social Anxiety Disorder After Trauma: A Case Report of Posttraumatic Growth?

**DOI:** 10.3389/fpsyt.2021.692637

**Published:** 2021-09-13

**Authors:** Verônica Hühne, Paula Vigne, Gabriela B. de Menezes, Leonardo F. Fontenelle

**Affiliations:** ^1^Obsessive, Compulsive, and Anxiety Spectrum Research Program, Institute of Psychiatry, Federal University of Rio de Janeiro (UFRJ), Rio de Janeiro, Brazil; ^2^D'Or Institute for Research and Education (IDOR), Rio de Janeiro, Brazil

**Keywords:** social anxiety, anxiety disorders, posttraumatic growth, traumatic event, trauma, resilience, case report

## Abstract

Posttraumatic growth (PTG) describes positive psychological change and improvement beyond one's previous functioning. It manifests as a change of self-perception, improvement in the relationship with others, and a better outlook on life. Despite consistent literature on the occurrence of PTG in healthy subjects, there is still a dearth of studies in people with pre-existing mental disorders, especially anxiety disorders. We report the case of a patient previously diagnosed with social anxiety disorder (SAD), whose symptoms remitted, and life view improved after a traumatic event, illustrating a case of PTG. The trauma shattered the patient's previous belief system, allowing the emergence of a new cognitive schema. Although PTG and symptom remission do not necessarily correspond to the same construct, we believe that these phenomena were related to each other in this case, probably because of a notable change in our patient's underlying belief system.

## Introduction

Social Anxiety Disorder (SAD) is a common anxiety disorder that, according to some estimates, can affect up to 13% of the population at some point during their lifetimes and 7.4% of the population in a given year (1). It is characterized by the fear of other people's judgment or scrutiny and avoidance of (or distress during) social situations (2). SAD is known for its early onset (usually in adolescence) and chronic course (3). At least in the United States, women seem to be more affected than men: one specific study suggested SAD to affect 14.2% of females and 11.8% of males during their lifetimes (1). Treating SAD is crucial because it is a risk factor for other mental disorders, including depression, substance use disorder, and other anxiety disorders; and a poor prognostic factor for comorbid disorders (2).

Posttraumatic growth (PTG), a concept introduced by Tedeschi and Calhoun in the 1990's, describes a positive psychological change (improvement surpassing someone's previous functioning level) after and because of a traumatic event. Different dimensions of growth are possible, including change in the perception of self and personal strength, perception of new possibilities, change in the relationship with others, a deeper appreciation of life, or spiritual change (4). According to the authors' own model for PTG, a specific event may be traumatic enough to rupture the individual's pre-existing cognitive schema and allows for new beliefs that positively embrace the traumatic event to flourish (5).

Tedeschi and Calhoun suggest that rumination is key to this process and typically present in the aftermath of trauma in the form of intrusive and unpleasant thoughts. In their model, though, once the individual starts processing the trauma, their ruminative thoughts help them give meaning to the event and build new cognitions (6). In literature, authors often compare the mechanism by which a traumatic event leads to PTG to an earthquake that destroys a city, leaving space for a stronger one, more resistant to new shocks, to be built (7, 8).

The relationship between PTG and Posttraumatic Stress Disorder (PTSD), two potential outcomes of a traumatic experience, is poorly understood. While some studies suggest that PTG and PTSD represent opposites of one single continuum (9), others claim that both conditions can coexist, even posing that some degree of posttraumatic stress is necessary for PTG (10). Although PTSD is relatively common [with a lifetime prevalence rate of 8%, almost four times more prevalent in women than men, and a 12-month prevalence rate of 3.7% (1)], there is still a dearth of studies exploring the impact of PTG in other mental disorders, including SAD. It is presently unknown, for instance, if the growth that may be experienced in the aftermath of trauma may comprise a positive effect on pre-existing mental disorders. In the current report, we present the story of a patient whose SAD remitted after a traumatic event.

## Case Report

Mr. A, a 57-year-old, single, administrative assistant sought treatment at our hospital almost 30 years ago after seeing a TV show about SAD. He argued being shy and reclusive since early childhood. Mr. A has always been ashamed of performing in front and interacting with other people. His symptoms progressively worsened as he grew up. By adolescence, he started presenting physical symptoms (like tremors, sweat, and heart palpitations) every time he exposed himself to social interactions. Mr. A had never dated until he was 31 years old when he had a brief romantic relationship strictly over the phone. He always lived with his mother.

Despite his qualifications and performance, Mr. A could not remain in the same job for more than 1 year, as he feared his coworker's judgment every time he had to eat in front of them. He also dreaded taking the bus due to being the center of attention while entering public transportations and quit the coding course because of the intense discomfort of having to type in front of people.

During the time Mr. A has been regularly seen in our service, he was treated for his social anxiety symptoms with clonazepam alone or in combination with multiple antidepressants, which were often interrupted because of intolerable side effects. No effective form of psychotherapy was available for him during the follow-up, and his conditions were managed exclusively with pharmacotherapy. Despite claiming he felt less anxious because of the treatment, Mr. A remained severely incapacitated and avoided going out and exposing himself. During this time, he never attained remission status. By the time he was 37 years old, he developed a depressive episode characterized by sadness, anhedonia, insomnia, and weight disturbances. After treatment with nortriptyline 150 mg/day (the only antidepressant he tolerated in a therapeutic dose), his depressive symptoms remitted. However, his social anxiety remained unchanged until age 49, when Mr. A went to a bank to withdraw money. While parking in an alley behind the building, armed assailants jumped out of a car and ambushed him. They hit him in the head with the gun and put him in the car. He was kept hostage for hours before being let go. He thought he would die. Of note, regardless of the emotional and financial injuries caused by the kidnapping, Mr. A never claimed or received any kind of insurance money or benefit because of the event.

Despite the hardship of the situation, Mr. A believes he has handled it well and repeats: “At the end of the day, I'm alive. I feel like a winner.” He denies ever displaying flashbacks of the event, affective distancing/numbing, hypervigilance, or avoidance of situations that remind him of the traumatic event. Further, despite remaining under the same pharmacotherapy scheme and not starting any effective form of psychotherapy during this whole process, his anxiety symptoms drastically improved after the traumatic event, and he finally attained remission status. “I used to care about what other people thought about me and feared their judgment badly. After the kidnapping, this sort of belief feels completely irrelevant,” he claims, “The kidnapping made me realize that the problem was inside of me. I can choose what affects me.”

Sometime after the trauma, Mr. A's salary was cut in half because of financial issues in the company he worked for. His symptoms did not worsen after this, and he says, “because of what happened to me, I'm much stronger. If I were like I was before, I wouldn't be able to handle it. But now I know that if being kidnapped didn't kill me, this won't either.” Still today, years after the traumatic event, Mr. A doesn't fear others' judgment. He eats in front of his coworkers without feeling heart palpitations or sweating and does not avoid social gatherings because of anxiety symptoms. Since the trauma, he has dated more often, has joined the gym, and has moved out of his mother's home. He likes going out and is happier while doing leisure activities. He says that “life is more colorful than before.” Table 1 depicts periodical assessments of the three dimensions of social anxiety symptoms (physical symptoms, fear and avoidance of social interactions) according to the Social Phobia Inventory (SPIN). As can be noted, there is a sustained improvement in Mr. A's SAD symptoms after the trauma.

**Table 1:** Change in anxiety symptoms.

		**17/12/2012**	**31/08/2015**	**26/08/2019**	**19/05/2021**
Spin (social phobia inventory)	Physiological symptoms	12	7	5	4
	Fear	15	7	7	6
	Avoidance	19	12	7	11
	Total	46	26	19	21

## Discussion

We reported the case of a patient with SAD whose anxious symptoms remitted and functioning improved after a trauma. He described greater internal strength (“I'm alive. I feel like a winner”), a greater appreciation of life (“life is more colorful than before”), was able to recognize new paths in life, and changed his priorities (“I used to care about what other people thought [but now] this feels completely irrelevant”). All of these are elements of PTG (4). In contrast to resilience, in which the subject returns to his previous functioning level after a traumatic event, the subject's functioning in PTG improves. It has been suggested that resilience may be a prerequisite for PTG (5). Still, a study in a population of people living with HIV did not find a higher prevalence of PTG in previously resilient people when compared to non-resilient individuals, suggesting that the pathways leading to each phenomenon may be different. One theory is that PTG could be developed parallel to resilience, without one interfering with the other (11).

Tedeschi and Calhoun suggest that PTG reconciles one's beliefs about being in control and life's predictability shattered by the trauma. According to them, the traumatic event would significantly challenge one's core beliefs and cause distress but result in cognitive reprocessing and ultimately personal growth. As seen in our patient, assumptions about people's judgment and its importance (core beliefs in SAD's cognitive schema) were dramatically changed after the event (“the kidnapping made me realize that the problem was inside of me. I can choose what affects me”). Another point of interest is the emotions related to the traumatic event and its consequences. The central hypothesis in literature is that growth happens through deliberate trauma processing that allows the individual to think about the event and what can be learned from it, thus deriving meaning from the trauma and developing new cognitions (6).

For instance, cognitive reappraisal, an emotion regulation strategy in which the individual changes the meaning and emotion attached to an event, was found to be related to PTG (7). Thus, experiences that elicit shame may be related to negative outcomes because one avoids thinking and talking about the events, likely inhibiting pathways that may lead to growth (12), including meaning-making (6). Mr. A felt proud and “like a winner” for having survived the kidnapping. It is also possible that the mechanisms through which trauma lead to improvement of the patient's social anxiety symptoms are the same as those involved in using mental imagery to modify the meaning of distressing autobiographical memories for SAD (13), to restructure dysfunctional thoughts, and to reappraise other people's judgment (14).

Traumatic events are occurrences, especially in large urban centers. Luz et al. in 2016 described a prevalence of 86% of traumatic events in Rio de Janeiro's and São Paulo's populations (15). Interestingly, while people who experience trauma and PTG seem to outnumber those that develop PTSD (12, 16), studies found that intense suffering may predict PTG (17) leading to the theory that growth is only possible after shattering previous beliefs and cognitive schema (18). There is evidence that spirituality, religious coping, and social support are also associated with PTG because they help one process the trauma and give it meaning (19). Other models tried to explain PTG and PTSD pathways by studying unique and shared predictors (20). For instance, a series of common predictors (such as lower levels of education, trauma severity, and non-Caucasian ethnicity) were positively associated with both conditions (12). Plus, there are also several reports of individuals in which both PTG and PTSD coexisted (10). For this reason, some authors suggest the existence of a more complicated relationship between positive and negative outcomes of trauma than just unidimensional (10).

Although several studies describe PTG in non-clinical and PTSD populations, only a few report PTG in patients with other psychiatric disorders. Mazor et al. (17) assessed the presence of PTG in patients with serious mental illness (mostly psychosis) who had PTSD symptoms. They tried to determine how the attribution of meaning and coping strategies influenced the PTG's formation process. They also evaluated whether PTSD symptoms had any influence on PTG's development. They found that lower posttraumatic stress total score was related to higher meaning in life questionnaire and higher coping self-efficacy total scores, which in turn were related to higher post-traumatic growth scores. For this reason, Mazor et al. suggested that avoidance symptoms, intrusive images, and hypervigilance would hinder trauma processing, thus preventing the necessary cognitive changes to lead to the development of PTG. However, these studies were conducted mainly with psychotic patients.

Also, in the field of serious mental illnesses, Jordan et al. published a systematic review on positive changes in patients and their families after a first psychotic break. Despite authors having observed mostly adverse outcomes of psychosis, like distress and reduced quality of life, positive results were also described, such as changes in interpersonal relationships and how they see the world (21). After this review, Jordan et al. conducted a qualitative study in a specialized center to evaluate positive experiences after a psychotic break. They found that these experiences happened similarly in frequency as in other types of trauma, like cancer and living with HIV. Patients described these positive outcomes as a change in behavior, their view of life, and an attempt to change past mistakes. These findings may be significant for other psychiatric conditions (22). The observation that PTG may occur in other mental disorders may help increase knowledge on the cognitive pathways toward improving anxiety disorders.

Unfortunately, a single case report is not enough to reach conclusions or generalize about a PTG's effect on SAD. However, it brings awareness to its possibility and prompts more studies on the topic. Keeping the limitations of a single case report in mind, a strength in our report is that we were able to follow the patient for a long time before and after the trauma, enabling us to know him and the course of his disorder reasonably well. Over the years, he had subtle symptoms fluctuations, but never a significant improvement as the one observed after the traumatic event. We were also able to document that his drastic amelioration has been maintained for years.

## Conclusion

There is a growing literature about positive experiences after a traumatic event, including the concept of PTG. Although several studies investigated the potential pathways from trauma to PTSD vs. PTG, there is still a lack of studies about PTG's impact on pre-existing psychiatric disorders, particularly anxiety disorders. We present a case report about a patient whose SAD symptoms remitted after a traumatic event due to his positive life view and cognitive schema change, characteristics of PTG. Although PTG and symptom remission do not necessarily correspond to the same construct, we believe that these phenomena were related to each other in our particular case, probably because of a unique change in Mr. A's underlying belief's system.

## Data Availability Statement

The original contributions presented in the study are included in the article/supplementary material, further inquiries can be directed to the corresponding author/s.

## Ethics Statement

The studies involving human participants were reviewed and approved by UFRJ—Instituto de Psiquiatria da Universidade Federal do Rio de Janeiro/ IPUB—UFRJ. The patients/participants provided their written informed consent to participate in this study. Written informed consent was obtained from the individual(s) for the publication of any potentially identifiable images or data included in this article.

## Author Contributions

VH contributed to the manuscript draft and research on the topic. PV contributed to the patient management and elaborated on patients' medical records. GM and LF provided clinical advice to PV on the case management and reviewed the case report and article as senior authors. All authors contributed to the article and approved the submitted version.

## Funding

This work was supported by the Conselho Nacional de Desenvolvimento Científico e Tecnológico (LF, grant number 302526/2018-8), Fundação de Amparo à Pesquisa do Estado do Rio de Janeiro (LF, grant number CNE E 26/203.052/2017), and the D'Or Institute of Research and Education (LF).

## Conflict of Interest

The authors declare that the research was conducted in the absence of any commercial or financial relationships that could be construed as a potential conflict of interest.

## Publisher's Note

All claims expressed in this article are solely those of the authors and do not necessarily represent those of their affiliated organizations, or those of the publisher, the editors and the reviewers. Any product that may be evaluated in this article, or claim that may be made by its manufacturer, is not guaranteed or endorsed by the publisher.
